# Natural course of intra-articular shifting bone marrow edema syndrome of the knee

**DOI:** 10.1186/1471-2474-9-45

**Published:** 2008-04-11

**Authors:** Nicolas Aigner, Roland Meizer, Gerd Petje, Elisabeth Meizer, Ashraf Abdelkafy, Franz Landsiedl

**Affiliations:** 1 Orthopaedic Hospital Vienna – Speising, Speisingerstrasse 109, 1130 Vienna, Austria

## Abstract

**Background:**

Intra-articular shift (migration) of bone marrow edema syndrome (BMES) is a very rare disease. Only a few cases have been reported thus far. The condition may cause the clinician to suspect an aggressive disease.

**Methods:**

We reviewed eight patients (four women and four men) with unilateral BMES located in the knee. The patients were aged 39 to 56 years (mean, 49.2 years). In all patients, bone marrow edema (BME) initially observed on magnetic resonance imaging (MR imaging) shifted within the same joint, i.e. from the medial to the lateral femoral condyle or the adjacent bone. Seven patients were given conservative therapy, including limited weight-bearing, for a period of three weeks after the initial detection of BMES, whereas one patient underwent surgical core decompression twice.

**Results:**

MR imaging showed complete restitution in 6 cases and a small residual edema in one case. A final control MR could not be obtained for one patient, who had no pain. A further patient had an avascular necrosis of the contralateral hip after 16 months. Improvement on MR imaging was correlated with the clinical outcome in all cases. All patients became asymptomatic after a mean period of 9 months (6–11).

**Interpretation:**

Intra-articular shifting BMES is a very rare condition. As the disease is self-limiting, conservative therapy may be recommended.

## Introduction

Bone marrow edema syndrome (BMES) is a common cause of severe bone and joint pain. The disease is usually self-limiting in nature and the symptoms resolve spontaneously over a period of 6, or occasionally 12 months. BMES primarily occurs in the femoral head, but also in the knee, foot, or less frequently at other locations. The patients complain of pain under stress and at rest, as well as a limited range of motion in the affected joint.

The exact pathogenesis of BMES remains unclear. Commonly discussed theories include thromboembolism, obstruction of arteriolar inflow or venous outflow, injury to the vessel wall secondary to vasculitis, altered lipid metabolism, and reduced fibrinolysis [1-4].

Bone marrow edema (BME) shows reduced signal intensity on T1-weighted MR images and increased signal intensity on T2-weighted MR images. No focal changes are identified and joint effusion is usually present [5]. The radiographs are initially normal but patients may subsequently develop severe osteopenia.

BMES is known to migrate between different joints of the same limb or different limbs (migratory osteoporosis, migrating BMES) [6]. However, to our knowledge, shifting of BMES between different compartments of the same joint has been previously reported only in a few cases [7-11]. The purpose of this retrospective study was to analyze the clinical course and MR images of this rare disease.

## Methods

Eight patients (four women and four men) aged 39 to 56 years (mean, 49.2 years), with intra-articular migratory BMES, were investigated retrospectively. The patients were retrieved from a collective of 50 consecutively diagnosed cases of painful BMES of the knee treated conservatively and by surgery. On the routinely performed MR investigation three months after the initial diagnosis, the location of the BME in the knee was found to have shifted to a different part of the knee. Patients with avascular necrosis (AVN) in the knee and other joints, and osteochondritis dissecans in a demarcated bone area; patients with BME secondary to osteoarthritis, mechanical stress or trauma; and patients with algodystrophy and corticosteroid medication at initial presentation were excluded. The average duration of pain had been 10 weeks (range, 6 to 18 weeks). Six patients (two women and four men) performed light sports (about 1 hour per week; one played soccer, two played tennis and three played golf). No patient presented with a history of BME in other joints than the knee. One had undergone surgery (partial medial meniscectomy) in the affected knee two years previously.

The laboratory data of the eight patients revealed mild lipid disorders in three patients, hyperglycemia in two, and elevated liver function tests in a further three patients (see additional file 1). Apart from these, no major disorders were found in blood parameters. Three patients smoked (5–30 cigarettes/day) and three reported regular intake of alcohol. The average body mass index (BMI) was 27.9 ± 7.3 (19.5 to 38.2); two patients were obese (BMI 33.8 and 38.2).

Plain anteroposterior and lateral radiographs under weight-bearing were available for every patient. They were found to be normal in five patients whereas two showed mild radiographic signs of osteoporosis and one showed minimal narrowing of the joint space in the bones affected by BME. Two patients presented with a mild varus deformity (hip/ankle angle 3° and 4° varus) of the leg axis.

MR imaging, including T1-weighted and T2-weighted fat suppressed (short tau inversion recovery) sequences, was performed before therapy. MR imaging was repeated every 12 weeks (8–14 weeks) until the MR imaging pattern normalized and the patients had no pain.

Treatment after the initial MR study consisted of a series of five infusions with 20 μg iloprost (Ilomedin^®^, Schering AG, Germany) in off-label use and in an inpatient procedure in seven patients, accompanied by 3 weeks of partial weight-bearing. Iloprost is a vasoactive prostacyclin analogue which dilates arterioles and venules, reduces capillary permeability and inhibits platelet aggregation [12]. One patient with BMES of the medial femoral condyle underwent core decompression of the affected condyle. Pain at rest and effort-induced pain were rated by the patients at each visit on a visual analogue scale (VAS) from 0 to 10 cm.

## Findings

**Figure 1:**
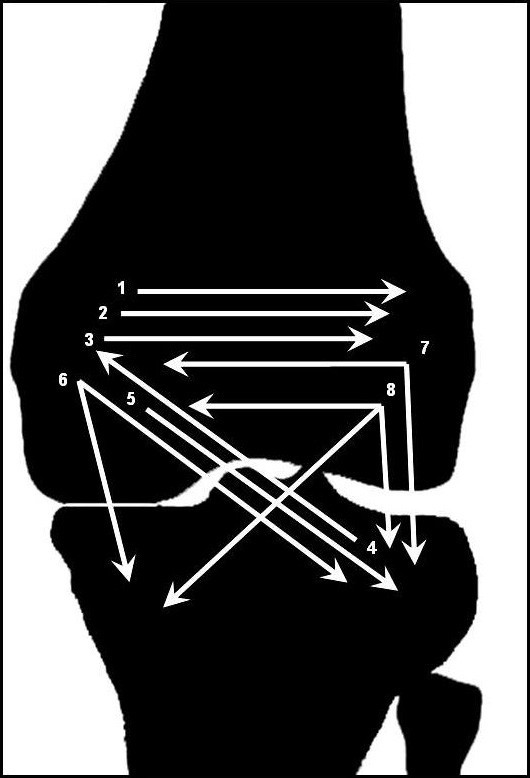
Shift of bone marrow edema.

At baseline MR imaging, six patients had BME in the medial femoral condyle, one patient in the lateral femoral condyle, and one in the lateral tibial plateau. The size of the BME was deemed large (50–100% of the femoral condyle or the tibial plateau) in five cases and medium (25–50%) in three cases. In addition, a mild effusion was detected in five of the eight cases. At the first MR imaging control performed 3 months after baseline, the BME had shifted horizontally from the medial to the adjacent lateral femoral condyle in three patients and vertically in two patients (one from lateral tibial plateau to the medial femoral condyle, and one vice versa). Two patients showed migration to the neighboring femoral condyle and to the lateral tibial plateau, one of these with an additional shift to the medial tibial plateau. In one patient a shift from the medial femoral condyle and the lateral as well as medial tibial plateau was observed (see additional file 1 and figure 1). The newly affected quadrants were large in one case, medium in five, and small in two cases. Healing of the primarily affected quadrants was seen only in one patient, partial regression in size in three patients, and no change of the BME pattern in the remaining four. Five patients stated that the location of the diffuse pain in the knee had changed since the beginning of the disease.

The patients continued the conservative therapy accompanied by reduction of their daily-life activities (seven patients), partial weight-bearing (two patients for a further 4 weeks), or intake of non-steroidal anti-phlogistic drugs (six patients) after the intra-articular migration of the BMES had been diagnosed. One patient with BMES in the medial femoral condyle underwent core decompression of the medial femoral condyle after BMES had been initially diagnosed; drilling of the lateral femoral condyle was performed 5 months after the first operation.

**Figure 2:**
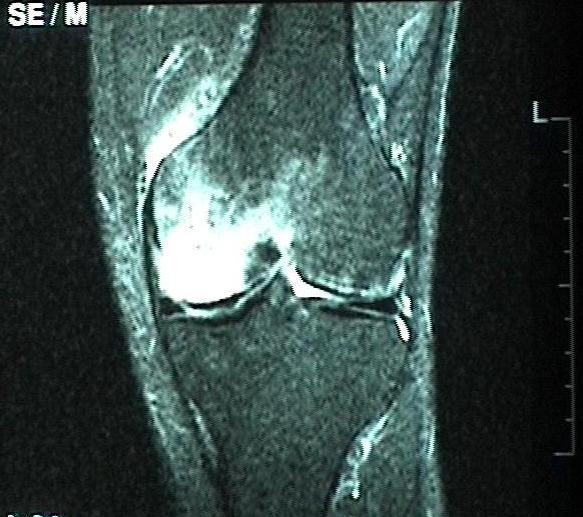
48-year-old man with a large BME in the medial femoral condyle not limited to the subchondral bone on the initial T2-weighted MR investigation (STIR).

**Figure 3:**
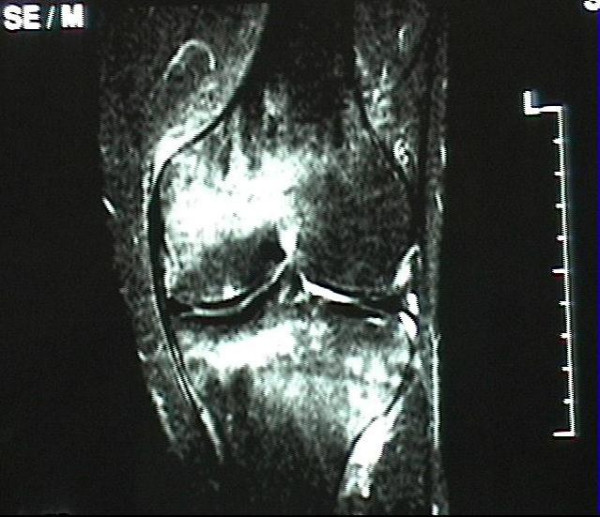
The MR investigation shows a shift of a large BME to the medial and the lateral tibial plateau, and a reduction in the size of the BME in the medial femoral condyle 3 months later.

**Figure 4:**
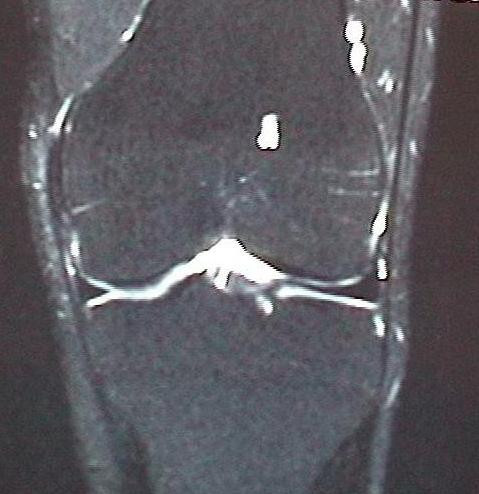
Complete normalization of the MRI signal pattern after 6 months of conservative treatment.

**Figure 5:**
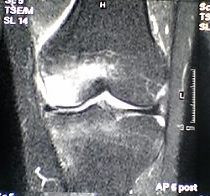
44-year-old woman with a extensive BME in the medial femoral condyle which is not limited to the subchondral area on the T2-weighted MR investigation (STIR).

**Figure 6:**
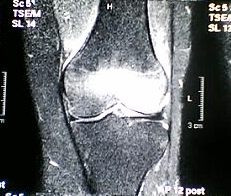
The MR image shows a shift of the large BME to the lateral femoral condyle, reduction in the size of the BME in the medial femoral condyle, and remission at the medial tibial condyle 4 months later.

**Figure 7:**
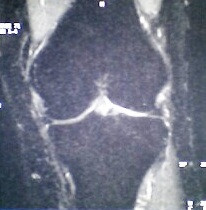
Complete normalization of the MR imaging signal pattern after 10 months.

The final MR investigation performed on average 8 months after baseline (range, 7–11 months) showed full resolution of BMES in 6 patients (figures 2 to 7). One patient had small residual edematous bone areas. No quadrant was newly affected. A final MR control could not be performed in one patient, who was free of pain.

The severity of effort-induced pain (VAS) was reduced from 7.5 (2.0–10.0) at baseline to 5.9 (2.4–7.9) after 3 months and to 0.6 (0–0.9) after the final examination. Pain at rest (VAS) diminished from 3.9 (1.5–7.8) to 2.8 (1.4–6.0) after 3 months and to 0 at the final follow-up. In one patient with BMES in the knee, avascular necrosis of the contralateral hip was evident within 16 months, which is being treated conservatively until the present time. All active patients were able to return to the same level of sports as that prior to commencement of their symptoms.

## Discussion

In 1959 Curtiss and Kincaid [13] reported three cases of young women who developed transient demineralization of the hip during the third trimester of pregnancy. Wilson et al. coined the term BME in 1988 after the introduction of MR imaging. It is controversially discussed as to whether BMES reflects a distinct self-limiting disease, a kind of reflex sympathetic dystrophy, or an early reversible form of osteonecrosis [1,10,14-16]. However, avascular osteonecrosis and BME are known to occur in different populations, which would militate against a common etiology. On the other hand, one of our patients with BMES in the knee showed avascular necrosis in the femoral head, which indicates a relationship between the two entities.

BMES is usually associated with a spontaneous onset of pain in the absence of prior trauma. Physical findings are minimal and laboratory values are usually normal or non-specific. Clinical improvement usually occurs over a period of 6, or occasionally 12 months. Joints of the lower limbs are much more commonly affected than those of the upper limbs.

Therapy options are conservative treatment (reduction of weight-bearing, analgesic and anti-inflammatory medication) and surgical treatment (core decompression) [17]. In our department we usually treat patients presenting with BMES with intravenous iloprost in combination with reduced weight-bearing, which appears to accelerate the natural course of the disease in many cases [16,18-22].

Many patients experience recurrence of BME in other joints, usually on the contralateral side. The term 'regional migrating osteoporosis' or 'regional migrating BMES' has been used in these cases. To our knowledge, a shift of BME within the different compartments of the same joint has only been described in a few case reports. As in our cases, migratory BMES had a benign course and improvement on the MR images was correlated with the clinical outcome in all patients [7-11].

In studies focusing on the knees, BMES was initially present in one femoral condyle and shifted to the contralateral femoral condyle or the tibia within a period of 2 to 4 months. In theory, this condylar shift could be due to a compensatory mechanism whereby the patient shifts weight-bearing to the unaffected painless side of the knee. However, this theory is rejected by some authors [9]. In a further study, BME in healthy volunteers with altered weight-bearing was less extensive in size and milder than that observed in our cases [23]. In another study it was found that typical BME caused by mechanical stress was mild, and located in the subchondral area. It had a linear or wedge-shaped appearance, whereas idiopathic BMES is characterized by large areas being affected, and not being limited to the subchondral bone [17]. Therefore, all three cases of BME with minimal degenerative changes in the joint and large edematous areas without limitation to the subchondral region were considered to be of primarily ischemic origin.

Finally, we observed normalization of the MR imaging pattern in all patients treated with conservative therapy alone, which would contradict a purely mechanical etiology of the BMES. While the presence of meniscal tears might lead to altered biomechanical loading, we do not believe this is responsible for the shift of edema since this kind of BME due to biomechanical stress would be limited to the subchondral bone. In addition, this situation would more likely explain a vertical shift, for instance from the medial femoral condyle to the medial tibial plateau, rather than a horizontal migration from medial to lateral.

Our patients did not show a subchondral linear abnormal signal pattern of this type, but had a large BME affecting at least 25% of the femoral condyle or the tibial plateau. Furthermore, no association was found between the size of BME and its migration. In those cases in which BME shifts to an adjacent quadrant, it appears that the origin is the primary affected quadrant. However, in cases of atypical migration (such as from proximal to distal or vice versa or diagonally), it is unclear whether the condition is a shifting or a new BME. The authors' thesis of the pathogenesis of intra-articular migratory BMES is a regional, diffuse, and therefore reversible ischemia of unknown origin affecting adjacent bones or parts of a bone. The non-simultaneous but consecutive affliction prolongs the total duration of pain than that seen in ordinary BMES. A progression to an AVN is not likely but cannot be entirely ruled out, depending on the extent of damage to the terminal vascular bed.

Intra-articular shifting (or migrating) BMES has also been reported in other joints of the lower limb. Fernandez-Canton [24] published 25 cases of BME in the foot with a one-year follow-up. Six of seven cases without complete restitution of the BME showed migration of BME to other bones that were initially normal, or to previously non-edematous areas of affected bones. Furthermore, the authors noted soft tissue edema in all 25 cases of BME of the foot, and joint effusion in 10 of 25 cases.

In addition, the diagnosis of BME migration may be suggested only after other causes of edema, such as avascular necrosis, subchondral insufficiency fracture, infection, and post-traumatic bone bruise, have been excluded. We continue to perform MR imaging studies every three months in patients whose symptoms do not abate under conservative therapy. Furthermore, we recommend early MR imaging diagnosis of other painful joints such as the hip, knee and ankle, especially on the contralateral side, to detect migrating BMES.

The vasoactive drug iloprost did not accelerate the course of the disease in patients with intra-articular shifting BMES as it does in non-shifting BMES [16,18-22] nor did it prevent shifting within the joint. Nevertheless, we recommend conservative therapy with partial weight-bearing and the administration of non-steroidal analgesic drugs rather than core decompression in these cases.

Limitations of the present report are the small number of patients and the retrospective nature of the study, which is due to the fact that this entity is very rarely seen. Further investigations are needed for a more comprehensive understanding of this disease.

## Conclusion

Intra-articular migration of a large BMES appears to be an aggressive phenomenon, but the disease has a benign course and is self-limiting in nature. We therefore recommend conservative therapy.

## Declaration of competing interests

The author(s) declare that they have no competing interests.

## Authors' contributions

NA: collection of data, first author. RM: collection of data. GP: collection of data. EM: analyzing of data, editing. AA: english editing. FL: supervision, editing.

## Pre-publication history

The pre-publication history for this paper can be accessed here:



## Supplementary Material

Additional file 1 Epidemiologic data and findings Click here for file
