# Supplementary material for: Natural course of intra-articular shifting bone marrow edema syndrome of the knee
Source: BMC Musculoskelet Disord. 2008 Apr 11;9:45. doi: 10.1186/1471-2474-9-45 (PMC2329633; doi:10.1186/1471-2474-9-45)
Supplement: Additional file 1 — Epidemiologic data and findings [file 1471-2474-9-45-S1.doc]

**Table 1: Epidemiologic data and findings**

| Pat. | Sex | Age | BMI | Treatment | Laboratory values | BME at MRI Baseline | BME in MRI after 3-4 months | BME in MRI after 6-8 months | BMI in MRI after 9-11 months |
| --- | --- | --- | --- | --- | --- | --- | --- | --- | --- |
| 1: JK | m | 56 | 33,8 | core decomp. 2 times | chol. 240, gluc. 134, ggt 99, aat 89 | m.f.c. | m.f.c., l.f.c. | restitution |  |
| 2: RH | f | 56 | 38,2 | conservative | chol. 210, gluc. 118 | m.f.c. | l.f.c. | not done | restitution |
| 3: SS | f | 44 | 19,5 | conservative |  | m.f.c. | m.f.c., l.f.c. | restitution |  |
| 4: ET | f | 39 | 20,5 | conservative | chol 210 | l.t.p. | l.t.p., m.f.c. | m.f.c. residual BME | restitution |
| 5: JS | m | 45 | 27,5 | conservative | ggt 262,  aat 64 | m.f.c. | m.f.c., l.t.p. | l.t.p.minimal residual BME |  |
| 6: GF | m | 48 | 28,1 | conservative |  | m.f.c. | m.f.c., m.t.p., l.t.p. | restitution |  |
| /: JG | m | 54 | 29,2 | conservative | ggt 49 | l.f.c. | l.f.c., m.f.c., l.t.p. | restitution |  |
| 8: IS | f | 52 | 27,0 | conservative |  | l.f.c. | l.f.c., m.f.c., l.t.p., m.t.p | restitution |  |

Abbreviations: m… male f… female chol… total cholesterol gluc … fasting glucose

ggt… gamma glutamyl transpetidase aat… alanine amino transferase

m.f.c… medial femoral condyle l.f.c… lateral femoral condyle

m.t.p… medial tibial plateau l.t.p… lateral tibial plateau
